# Disorganization of Small‐World Functional Brain Networks in First‐Episode, Treatment‐Naïve Adolescents With Major Depressive Disorder

**DOI:** 10.1002/brb3.70323

**Published:** 2025-02-11

**Authors:** Song Cheng, Henry H. Y. Tong, Chaoqing Zhang, Lingyu Jian, Junjun Ji, Ting Li, Yu Wang, Junfeng Li, Kefeng Li

**Affiliations:** ^1^ Department of Radiology Heping Hospital Affiliated to Changzhi Medical College Changzhi Shanxi China; ^2^ Changzhi Key Lab of Functional Imaging for Brain Diseases Heping Hospital Affiliated to Changzhi Medical College Changzhi Shanxi China; ^3^ Department of Radiology Tongde Hospital of Zhejiang Province Hangzhou Zhejiang Province China; ^4^ Centre for Artificial Intelligence Driven Drug Discovery, Faculty of Applied Sciences Macao Polytechnic University Macao China; ^5^ Department of Psychiatry Changzhi Mental Health Center Changzhi Shanxi China

**Keywords:** adolescent, depression, first‐episode, rs‐fMRI, small‐world, topological organization, treatment‐naïve

## Abstract

**Background and Aims:**

Adolescent major depressive disorder (MDD) is prevalent globally but often goes unnoticed due to differences in symptoms compared to adult criteria. Analyzing the brain from a network perspective provides new insights into higher‐level brain functions and its pathophysiology. This study aimed to investigate changes in the topological organization of functional networks in adolescents with first‐episode, treatment‐naïve MDD.

**Method:**

The study included 23 adolescents with depression and 27 matched healthy controls (HCs). Resting‐state functional MRI (rs‐fMRI) was conducted, and whole‐brain functional networks were constructed. Graph theory analysis was used to evaluate network topological properties. A machine‐learning multivariate diagnostic model was developed using network metrics associated with depression severity.

**Results:**

Both the MDD and HC groups displayed small‐world topology, with male MDD patients showing reduced global clustering efficiency (Cp). The nodal Cp (NCp) and local efficiency (NLE) in the bilateral pallidum were significantly positively correlated with depression severity. In contrast, nodal efficiency (NE) in the left medial orbital superior frontal gyri (ORBsupmed) showed a negative correlation with disease severity. A machine‐learning multivariate model using regional network topological features produced an AUROC of 0.71 (95% CI: 0.54–0.92) and an F1 score of 0.65, successfully differentiating adolescent MDD from HCs.

**Conclusion:**

Our findings suggest disruptions in small‐world topology in both global and local brain networks in adolescent depression. These abnormal nodal properties may serve as novel neural markers of the disorder.

## Graphical abstract

This study employs resting‐state functional magnetic resonance imaging (fMRI) to recruit adolescents diagnosed with major depressive disorder (MDD) and healthy controls (HCs), collecting clinical data such as disease duration and symptom severity. By constructing and comparing the brain network topological properties of the two groups, significant alterations in nodal characteristics (e.g., degree centrality, efficiency) in key brain regions of MDD patients were identified, revealing abnormalities in the functional brain network. Further analysis demonstrated a significant correlation between the key nodal characteristics and disease severity. Moreover, a multivariate model based on small‐world network features was developed using the random forest algorithm to predict MDD diagnosis, providing new network biomarkers and diagnostic tools for clinical use.

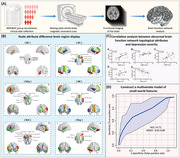

**Abbreviation**
ACG: anterior cingulate and paracingulate gyri
ALFF: amplitude low‐frequency fluctuation
BC: betweenness centrality
Cp: clustering efficiency
DC: nodal degree centrality
DCG: median cingulate and paracingulate gyri
DMN: default mode network
Eglob: global efficiency
Eloc: local efficiency
FC: functional connectivity
HAMD‐17: Hamilton‐17 Depression Rating Scale
HES: heschl gyrus
ICA: independent component analysis
IFGoperc: opercular part of inferior frontal gyrus
IFGtriang: inferior frontal gyrus, triangular part
INS: insula
Lp: characteristic path length
MDD: major depressive disorder
MRI: magnetic resonance imaging
NCp: nodal clustering efficiency
NE: nodal efficiency
NLE: nodal local efficiency
NLP: nodal shortest path length
ORBinf: orbital part of inferior frontal gyrus,
ORBsupmed: medial orbital superior frontal gyrus
PAL: pallidum lenticular nucleus
PUT: putamen lenticular nucleus
ReHo: regional homogeneity
ROL: rolandic operculum
rs‐fMRI: resting‐state functional MRI
SAN: salience network
SFGmed: medial superior frontal gyrus
SMG: supramarginal gyrus
STG: superior temporal gyrus
WHO: World Health Organization

## 1 Introduction

Major depressive disorder (MDD) is a prevalent and complex mental illness, primarily marked by persistent sadness and low mood (H. Yu et al. 2018). The World Health Organization (WHO) ranks MDD as the third most prevalent illness, after cancer and cardiovascular diseases. In 2015, MDD affected around 350 million people globally, contributing to 10% of the total disease burden worldwide (Smith 2014; Li et al. 2020). Moreover, the COVID‐19 pandemic is anticipated to significantly increase the incidence of MDD in the coming years (Li et al. 2020).

Adolescence is a critical phase of physiological and psychological development, where individuals are particularly vulnerable to mental disorders due to emotional, social, and substance‐related stressors (M. Yu et al. 2019; Gunnell, Kidger, and Elvidge 2018). Adolescent MDD exhibits more atypical clinical symptoms compared to adult MDD and is associated with higher rates of recurrence and disability. About 60% of adolescents with depression attempt suicide, making it a leading cause of death in this age group (Bella, Fernández, and Willington 2010). Adolescent MDD is frequently accompanied by cognitive impairments, including memory loss and impaired executive function, significantly affecting quality of life and academic performance (Baller et al. 2021; Holt et al. 2016).

Most research on MDD has focused on adults, with fewer studies addressing adolescent MDD (Uher et al. 2014). Recent studies have began exploring the neural basis of adolescent MDD. Cullen et al. (2014) were the first to identify abnormalities in resting‐state functional connectivity (FC) of the amygdala in adolescent MDD, opening new avenues for research. More recently, Jamieson et al. (2022) and Willinger et al. (2024) found altered effective connectivity in adolescent MDD, particularly in the salience and default mode networks (DMNs). These findings suggest that alterations in these brain networks play a crucial role in the pathophysiology of adolescent MDD.

Currently, the diagnosis of adolescent MDD primarily relies on clinical experience and depression scale screenings (Uher et al. 2014). However, depression scales can be subjective, making it essential to explore objective biomarkers for MDD's prevention and treatment.

Resting‐state functional magnetic resonance imaging (rs‐fMRI), a noninvasive tool, has been increasingly utilized in the diagnosis and research of neuropsychiatric disorders in recent years (Luczynski, Moquin, and Gratton 2015; Liston et al. 2006; Gourley Swanson, and Koleske 2013; Dias‐Ferreira et al. 2009). Rs‐fMRI measures the changes in blood oxygen levels to assess brain function and network connectivity (Fateh et al. 2019). Studies have shown that MDD patients exhibit abnormal FC in regions like the prefrontal cortex (PFC) and hippocampus (Qiu and Li 2018; Chen et al. 2020). For instance, Willinger et al. (2024) reported reduced effective connectivity between the salience and DMNs in adolescent MDD, suggesting that this disruption may impair emotional regulation and self‐referential processes, both critical to MDD's pathophysiology. In addition, research suggests that whole‐brain fMRI signals may serve as potential biomarkers to distinguish MDD from other mental disorders, offering valuable insights for treatment (Kraus et al. 2020; Yang et al. 2021).

Earlier research primarily focused on local brain FC, using methods like regional homogeneity (ReHo), amplitude of low‐frequency fluctuation (ALFF), FC, and independent component analysis (ICA). However, growing evidence suggests that MDD arises from emotional and cognitive dysfunction, with abnormal FC between brain regions playing a central role (Kaiser et al. 2015; Mulders et al. 2015; Zhao et al. 2014). Alterations in local brain regions alone cannot fully explain the widespread abnormalities in brain function.

Despite the increasing use of graph theory analysis in adult MDD research, findings have been somewhat inconsistent. Some studies report changes in the small‐world properties of whole‐brain networks in adult MDD, while others fail to replicate these findings (Nelson and Bonner 2021). These inconsistencies may stem from factors like participant age, disease stage, or treatment history. Thus, investigating whether untreated adolescent MDD patients show similar topological network changes is crucial. Although studies on adolescent MDD have reported abnormalities in dynamic FC (Zheng et al. 2022), these findings do not fully reveal the global network characteristics of adolescent MDD.

This study aims to systematically analyze whole‐brain functional networks in adolescent MDD to clarify its neural basis. Given the rapid brain development during adolescence, distinct from the adult stage, this study focuses on first episode, drug‐naïve adolescent MDD patients, exploring the small‐world properties of their functional brain networks via graph theory analysis. The primary goal is to clarify changes in global and local network efficiency across specific brain regions and to explore the relationship between these properties and depression severity. This investigation aims to provide new evidence for early intervention and potential treatment targets in adolescent MDD. We hypothesize that the small‐world properties of brain networks in adolescent MDD patients will change and that these changes will correlate with symptom severity.

## 2 Methods

### 2.1 Study Participants

In this study, the patient group comprised 23 drug‐naïve adolescents aged 10–19 years who were first diagnosed with MDD at Changzhi Mental Health Center between November 2020 and January 2022. This group included 6 boys and 17 girls. The healthy control (HC) group consisted of 27 age‐ and gender‐matched healthy volunteers (11 boys and 16 girls), recruited through WeChat and advertisements. The study was approved by the Ethics Committee of Changzhi Medical College and registered with the Chinese Clinical Trial Registry (Registration No.: ChiCTR2000038210).

To ensure the welfare of adolescent participants, we implemented strict measures, including continuous monitoring by clinical psychologists, regular communication with participants and guardians, support and referral for psychological distress, and adherence to ethical guidelines with informed consent and confidentiality.

#### 2.1.1 Inclusion and Exclusion Criteria

The inclusion criteria were as follows: (1) The case group met the symptomatology, disease severity, and course criteria for the diagnosis of depression of the Chinese Classification and Diagnostic Standards of Mental Disorders, version 3 (CCMD‐3), and the DSM‐V, and had a Hamilton‐17 Depression Rating Scale (HAMD‐17) score of 7 or higher; (2) The patients had not received any treatments; (3) the HC group had no mental disorder, such as depression or epilepsy (their HAMD‐17 score was < 7), and the MDD group had no mental disorder other than depression; (4) neither group had any physical diseases or a history of alcohol or drug abuse; (4) the first‐degree relatives of patients and HCs had no relevant medical history; (5) patients and HCs were right‐handed; (6) neither patients nor HCs had any contraindications related to MRI scanning; and (7) patients and HCs provided their informed consent to participate in the study, were willing to participate in the groups, and could actively cooperate.

The exclusion criteria were as follows: (1) A history of alcohol and drug abuse (antipsychotic‐related drugs, etc.), (2) epilepsy or bipolar depression, (3) previous history of brain injury or brain surgery, (4) pregnancy in female participants, and (5) other MRI scan contraindications.

### 2.2 MetaData Collection

The metadata from the enrolled participants, including age, sex, height, weight, occupation, ethnicity, left‐ or right‐handedness, years of education, past medical records, and family medical history, from the enrolled participants were collected by professionally trained physicians. All enrolled patients were diagnosed with a unipolar depression episode by two professional psychiatrists, and all participants were assessed for depression using the HAMD‐17.

### 2.3 MRI Image Acquisition

#### 2.3.1 Image Scan Parameters

Whole‐brain rs‐fMRI scans were performed using a Siemens 3.0 T MAGNETOM Skyra MRI scanner with a 32‐channel head coil (Germany). All imaging data were acquired by the same 32‐channel RF coil scanner to increase the image quality and improve the image signal‐to‐noise ratio. Whole‐brain data were collected parallel to the anterior and posterior joint lines. The following sequences were used: (1) Routine sequence scanning: T1WI, T2_FLAIR, excluding those with organic lesions. (2) The fast gradient echo sequence was used to collect the structural images required for data analysis (3D‐T1 weighted structural phase). The parameters were as follows: TR (repetition time) = 2530 ms, TE (echo time) = 2.98 ms, flip angle = 90°, number of layers = 192, floor thickness = 1 mm, planar voxel resolution = 1 mm × 1 mm × 1 mm, and vision (field of view [FOV]) = 256 × 256 mm^2^. The acquisition time was 6 min and 3 s. (3) Functional images were collected using an echo planar imaging (EPI) sequence with the following parameters: 3.5 mm layer thickness, layer spacing = 3.5 mm, number of layers = 32, echo time = 30 ms, repetition time = 2000 ms, flip angle = 90°, field = 224 × 224 mm^2^, matrix size = 64 × 64, and plane voxel resolution = 3.5 mm. For each participant, 240 functional images were collected, with a scanning duration of 8 min and 6 s.

#### 2.3.2 Image Scanning Method

Participants lay flat on the MRI scanning bed with their eyes closed and their head fixed with a foam head pad. Noise‐canceling earplugs were given to the participants to reduce the MRI noise, and they were instructed to avoid thinking as much as possible.

#### 2.3.3 Preprocessing and Analysis of MRI Data

##### 2.3.3.1 rs‐fMRI Data Preprocessing

The rs‐fMRI data were preprocessed using GRETNA (Graph Theoretical Network Analysis) 2.0 software. The preprocessing process included (1) the conversion of the original DICOM data file into NIfTI format; (2) the removal of the first 10 time‐point images; (3) time‐layer correction; (4) head dynamic correction; (5) space standardization; (6) space smoothing: the full width and half height core was smoothed by 4 mm; (7) linear drift; (8) regression covariate analysis: whole‐brain signal, CSF signal and brain white matter signal; and (9) time bandpass filtering (0.01–0.08 Hz): filtering high‐frequency and ultralow‐frequency noise signals.

#### 2.3.4 Build the Functional Connection Matrix

Brain FC matrices were constructed using GRETNA 2.0 software. First, a participant's AAL (automated anatomical labeling) map was used to divide the brain into 90 brain regions, obtain the average time series of each brain region, calculate the Pearson correlation coefficients between the 90 brain regions, and then transform these correlation coefficients by Fisher *Z* to obtain a symmetric 90 × 90 correlation coefficient matrix.

#### 2.3.5 Analysis of Brain Functional Networks

We used GRETNA 2.0 to compute global and node‐level properties of the brain network, applying a sparsity range of 0.10–0.50 with a 0.01 interval. In addition, we calculated the area under the curve (AUC) for each network matrix. The sparsity threshold range of 0.10–0.50 for constructing the functional brain network was based on prior validated recommendations from several neural network studies (He et al. 2009; Bullmore and Sporns 2009).

To further validate the rationale for selecting the sparsity threshold, we analyzed the results at different sparsity thresholds, specifically including 0.1, 0.2, 0.3, 0.4, and 0.5. Under these different sparsity thresholds, we constructed functional brain networks and calculated key network indicators, including global efficiency (Eglob), local efficiency, and small‐world coefficient. The results showed that within the selected range of 0.1–0.5, the network characteristics remained relatively stable, indicating that the network structure and FC features at these sparsity thresholds were consistent, thereby further supporting the rationale for our choice of sparsity. Three node parameters were used together to test the centrality of each node in the network, including betweenness centrality (BC), nodal degree centrality (DC), and nodal efficiency (NE). The calculated differential brain regions were visualized using BrainNet Viewer (https://www.nitrc.org/projects/bnv) software. The global network efficiency indicators included Eglob and Eloc (Local efficiency). (2) The small‐world parameters included Cp (Clustering efficiency), Lp (Characteristic path length), the standardized clustering coefficient (gamma, γ), normalized feature path length (lambda, λ), and small‐world scalar (sigma, σ).

### 2.4 Statistical Analysis

General clinical data were analyzed by SPSS 22.0 software. Continuous variables such as age, height, and weight were compared by two independent‐sample *t* tests and are expressed as the mean ± SD. Multiple comparisons of the results were verified using the Bonferroni correction. Categorical data were compared by the chi‐square test. The differences in the global topological properties between the groups were evaluated using the AUC. The AUC values can provide a total scalar of the topological properties of the brain networks without the selection of a single threshold, and *p *< 0.05 was considered significant. Pearson's correlation analysis was performed between global and node attribute measures of patients with MDD and the Hamilton Depression Scale scores. The network attributes correlated with HAMD‐17 scores were selected to differentiate the depression group from the control group using the random forest (RF) algorithm.

## 3 Results

### 3.1 Participant Information

There were no differences in sex, age, or education level between the MDD and control groups (*p *> 0.05) (Table 1).

**TABLE 1:** The comparison of the general data between the MDD group and the HC group.

Clinical variables	MDD (*n* = 23)	HC (*n* = 27)	*T* value/*χ* ^2^	*p* value
Age (year)	15.2 ± 1.56	15.6 ± 2.17	0.77	0.45
Gender (male/female)	6/17	11/16	0.37	0.46
Educational level (years)	8.17 ± 1.57	8.56 ± 2.17	0.77	0.46
Good hand (right, left)	Right	Right	—	—
HAMA‐17 score	16.3 ± 5.44	1.07 ± 1.56	−12.98	0.00

*Note*: Age, educational level, and HAMA‐17 score were compared using two independent‐sample *t* tests and are expressed as mean ± standard deviation (SD). Gender was compared using the chi‐square test.

Abbreviation: HAMD‐17, Hamilton‐17 Depression Rating Scale.

### 3.2 Analysis of Global Topological Organization

**FIGURE 1:**
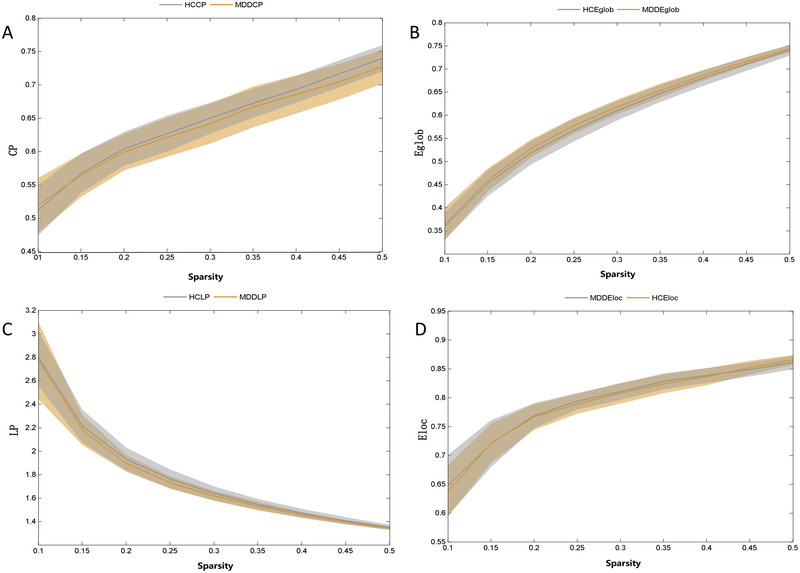
Group comparison of global network topological properties between adolescents with depression (MDD) and healthy controls (HC). (A) Cp: clustering efficiency; (B) Eglob: global efficiency; (C) LP: characteristic path length; (D) Eloc: local efficiency.

**FIGURE 2:**
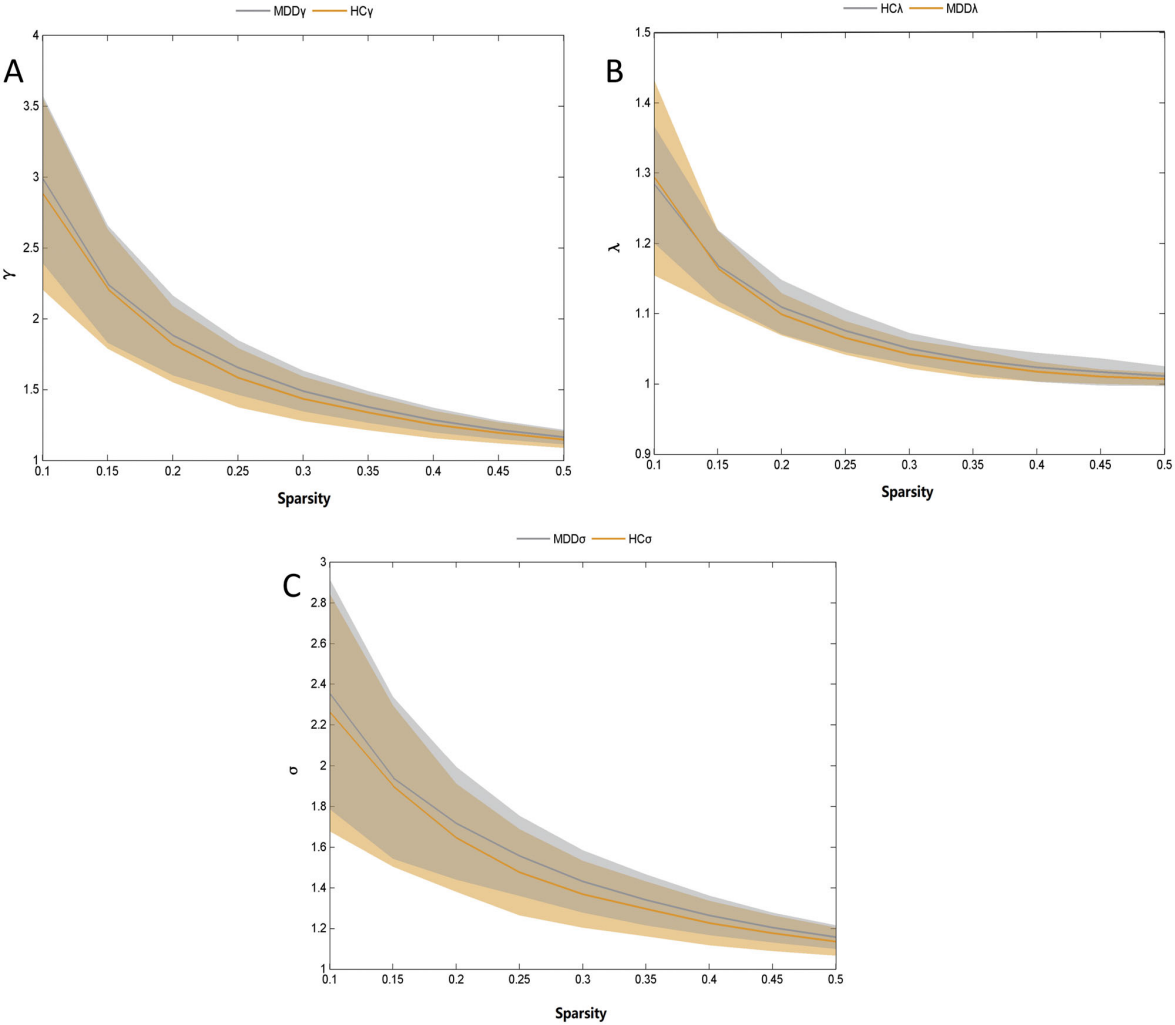
Group comparison of global network small‐world parameters between adolescents with depression (MDD) and healthy controls (HC). (A) *γ*: standardized clustering coefficient; (B) *λ*: standardized characteristic path length; (C) *σ*: small world scalar.

**FIGURE 3:**
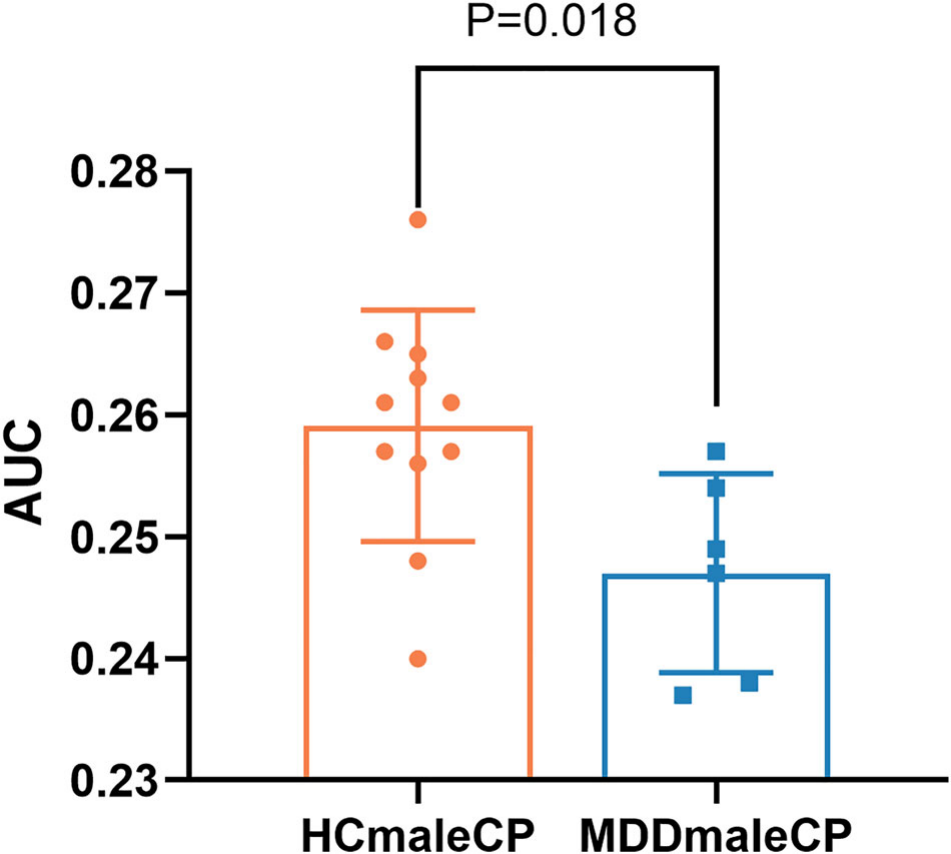
The global clustering efficiency (Cp) in male adolescents with MDD was significantly reduced compared with that in male controls. Data are expressed as the mean ± standard deviation (SD), *n* = 11 for HC_male_ and 6 for MDD_male_. Statistical analysis was performed using two independent samples *t*‐test, *p* <0.05. AUC, area under the curve; HC, healthy controls; MDD, major depressive disorder.

**FIGURE 4:**
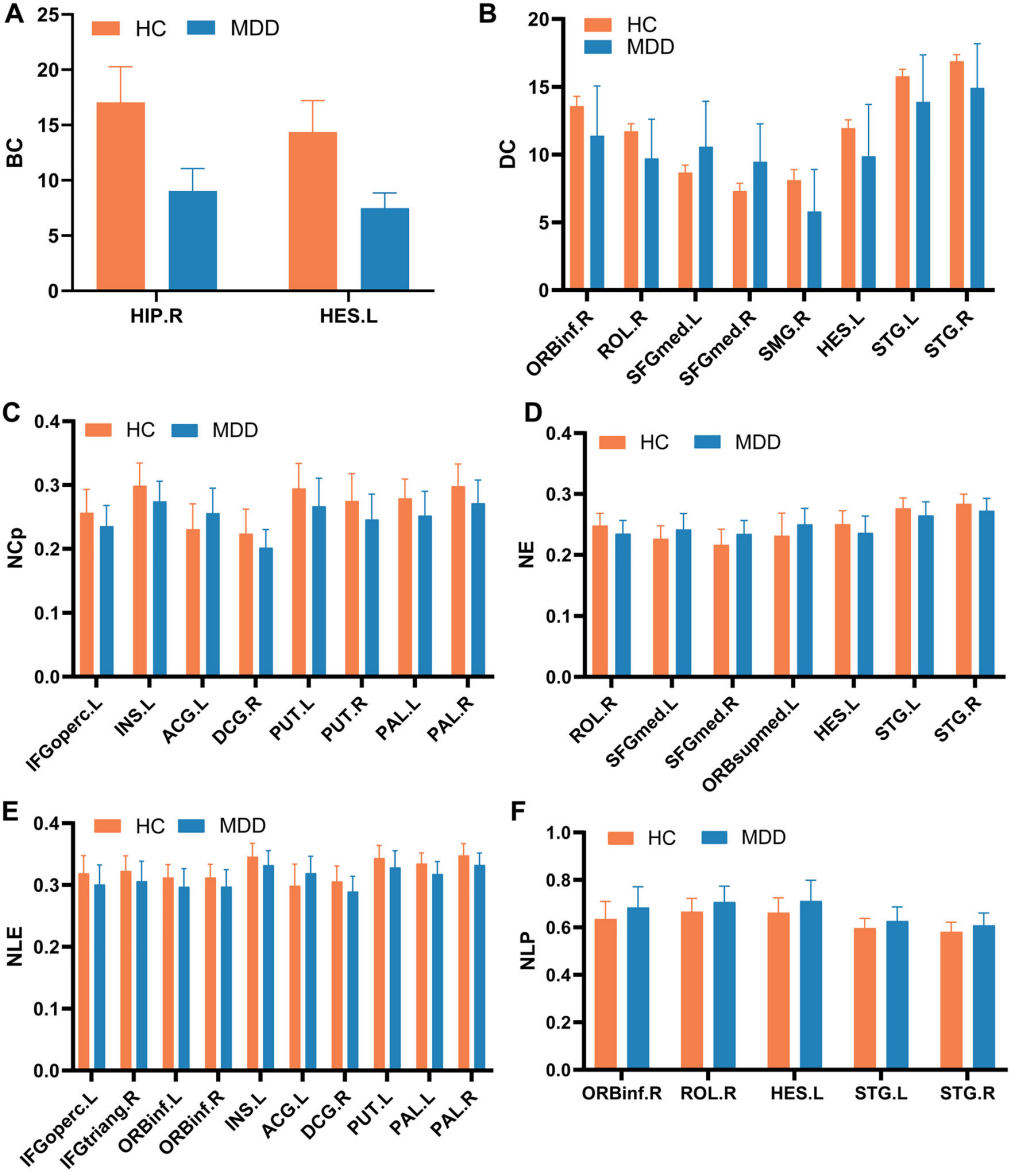
Comparison of nodal topological properties between the adolescents with depression (MDD) and the healthy controls (HC). Data are expressed as the mean ± standard deviation (SD), *n* = 27 for HC and 23 for MDD. Statistical analysis was performed using two independent samples *t*‐test, *p* < 0.05. BC, betweenness centrality; DC, nodal degree centrality; HC, healthy controls; MDD, major depressive disorder; NCp, nodal clustering efficiency; NE, node efficiency; NLE, node local efficiency; NLp, node shortest path length.

Overall, both the MDD group and the HC group had small‐world topology in the brain functional networks (*γ* = *C*_real_/*C*_random_ > 1, *λ* = *L*_real_/*L*_random_ > 1, and *σ* = *γ*/*λ* > 1). The global index analysis showed that *E*_glob_, *E*_loc_, *C*_p_, *L*_p_, *γ*, *λ*, and *σ* were not significantly different between the two groups (*p *> 0.05) (Table 2; Figures 1 and 2). However, when males and females were analyzed separately, Cp in males with MDD was significantly lower than that of the males in the control group (*p *< 0.05) (Figures 3 and 4).

**TABLE 2:** Comparison of global attribute area under the curve (AUC) between the major depressive disorder (MDD) group and the healthy controls (HC) group.

Global attributes	MDD	HC	*T* value	*p* value
Cp	0.26 ± 0.01	0.26 ± 0.01	1.08	0.29
Lp	0.69 ± 0.02	0.70 ± 0.03	1.15	0.26
Eloc	0.32 ± 0.00	0.32 ± 0.01	−0.52	0.61
Eglob	0.24 ± 0.01	0.24 ± 0.01	−1.38	0.17
*γ*	0.66 ± 0.08	0.64 ± 0.08	−0.83	0.41
*λ*	0.43 ± 0.01	0.43 ± 0.01	0.81	0.42
*σ*	0.61 ± 0.08	0.59 ± 0.08	−0.94	0.35

*Note*: Data are expressed as mean ± standard deviation (SD). Statistical analysis was performed using a two independent samples *t*‐test, with *p* < 0.05.

Abbreviations: Cp, clustering coefficient; Eglob, global efficiency; Eloc, local efficiency; Lp, characteristic path length; *γ*, standardized clustering coefficient; *λ*, standardized characteristic path length; *σ*, small world scalar.

### 3.3 Altered Regional Topological Organization

**FIGURE 5:**
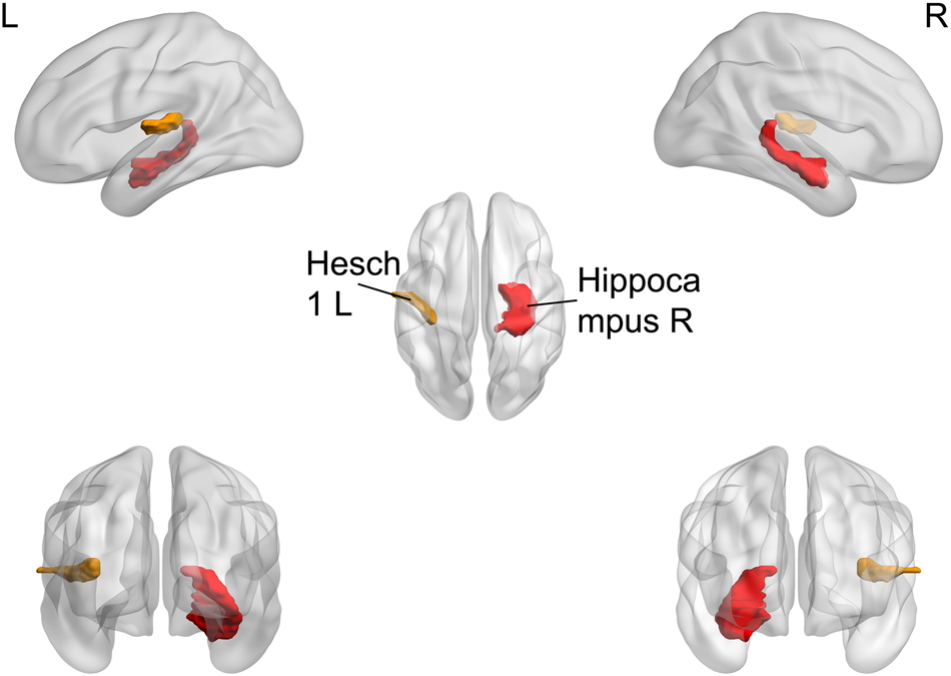
Brain regions with significant differences in the betweenness centrality (BC) between the major depressive disorder (MDD) group and healthy controls (HC). HC, healthy controls, L, left, MDD, major depressive disorder, R, right.

**FIGURE 6:**
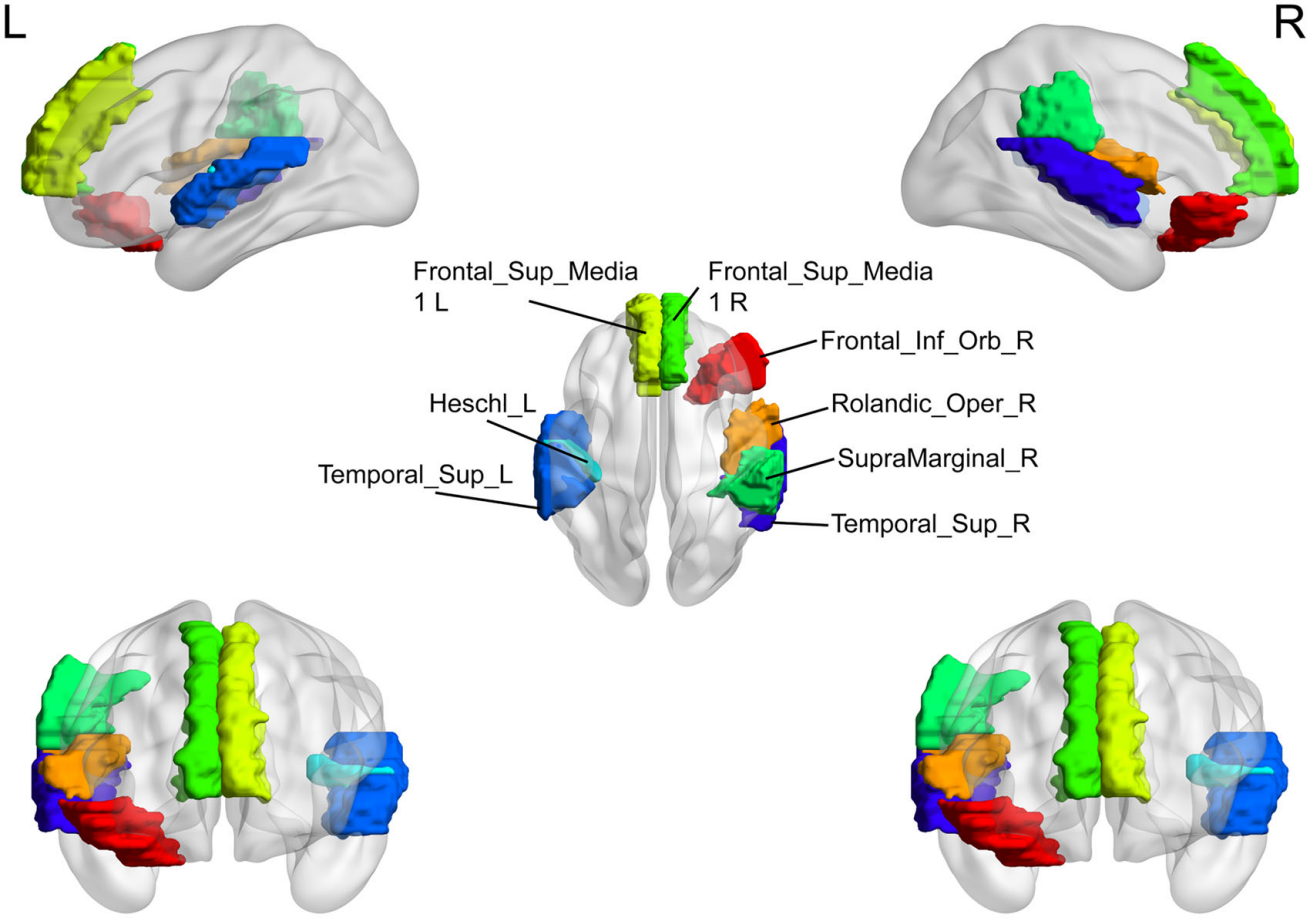
Brain regions with significant differences in the nodal degree centrality (DC) between the major depressive disorder (MDD) group and healthy controls (HC). HC, healthy controls, L, left, MDD, major depressive disorder, R, right.

**FIGURE 7:**
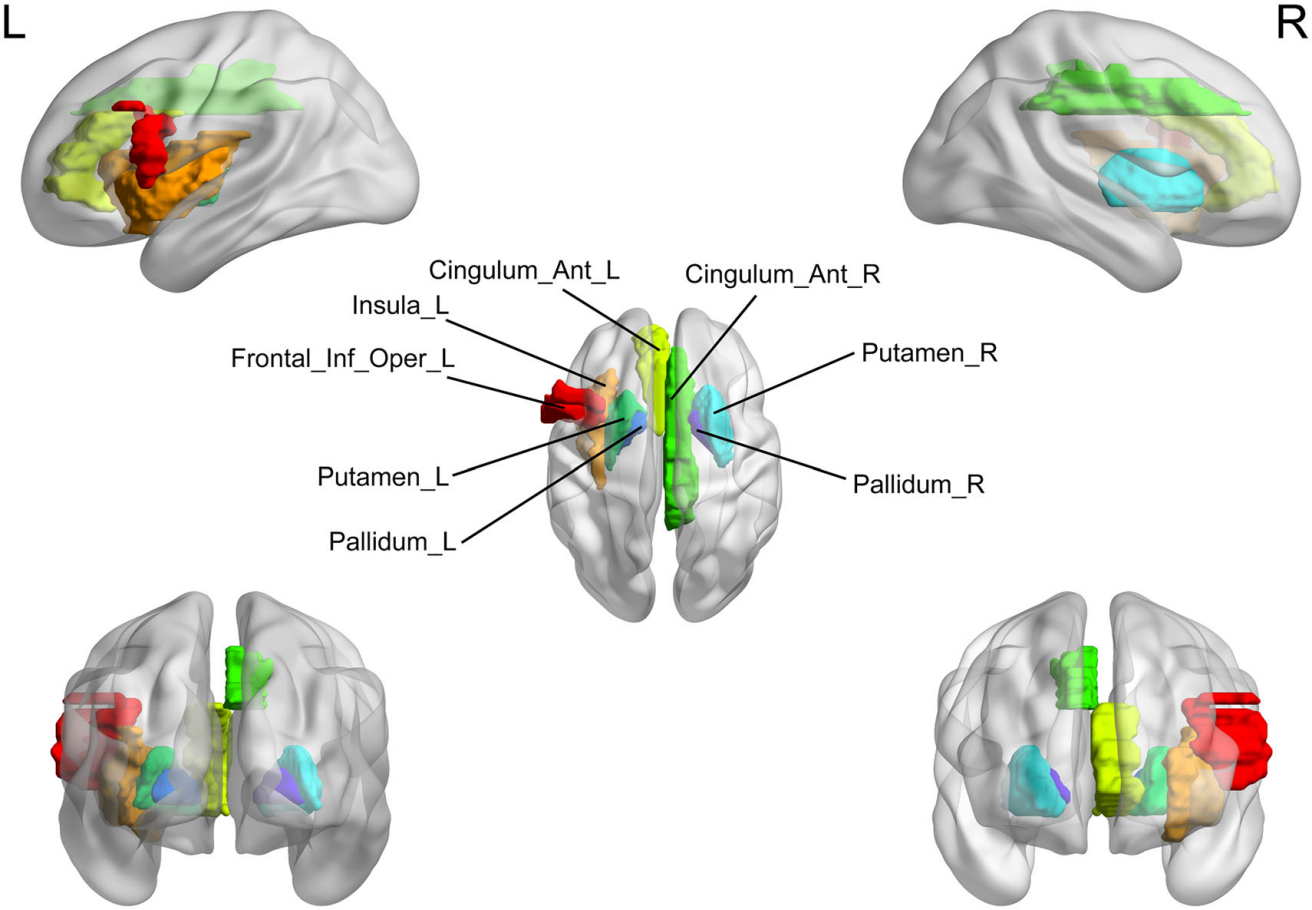
Brain regions with significant differences in the nodal clustering efficiency (NCp) between the major depressive disorder (MDD) group and healthy controls (HC). HC, healthy controls; L, left; MDD, major depressive disorder; R, right.

**FIGURE 8:**
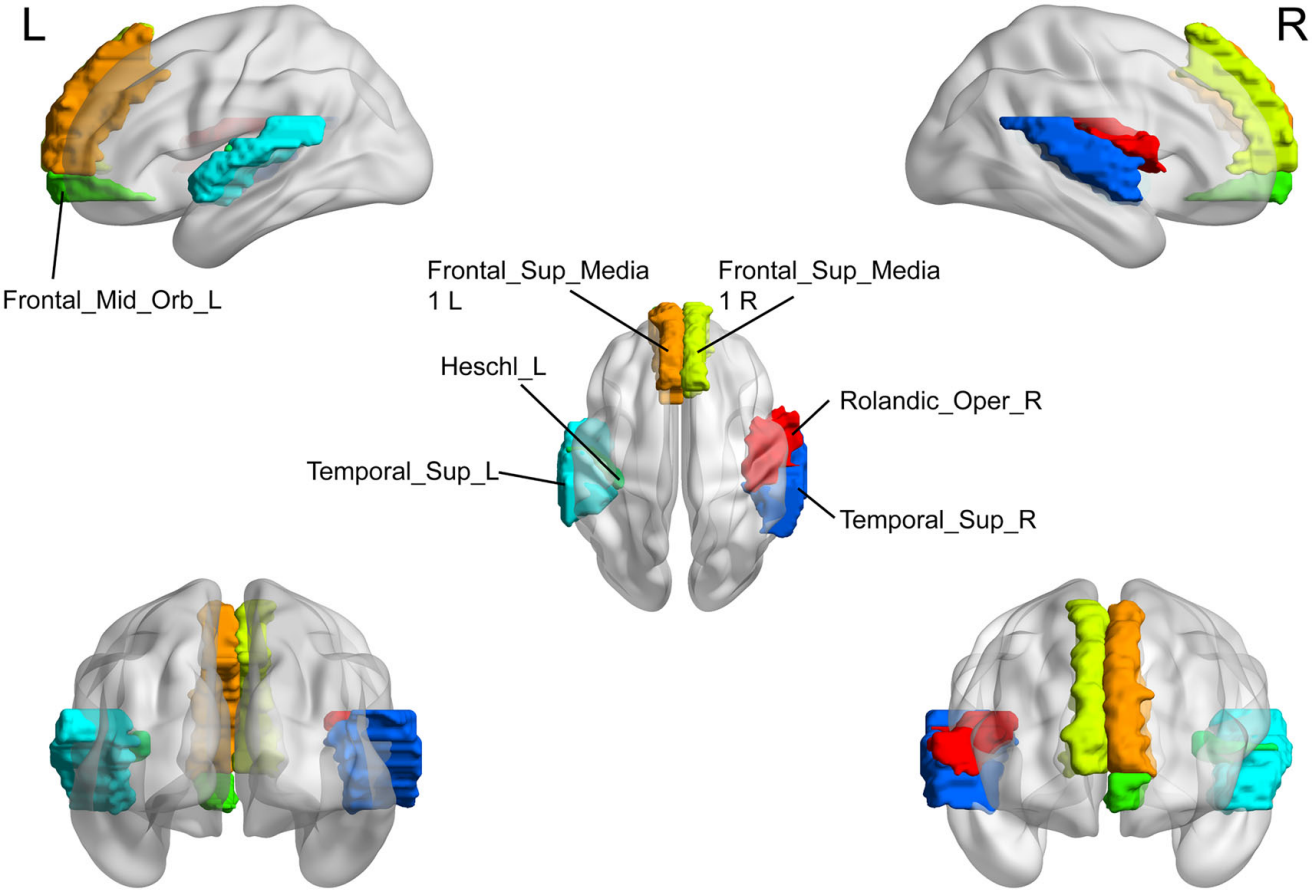
Brain regions with the significant differences in the nodal efficiency (NE) between the major depressive disorder (MDD) group and healthy controls (HC). HC, healthy controls; L, left; MDD, major depressive disorder; R, right.

**FIGURE 9:**
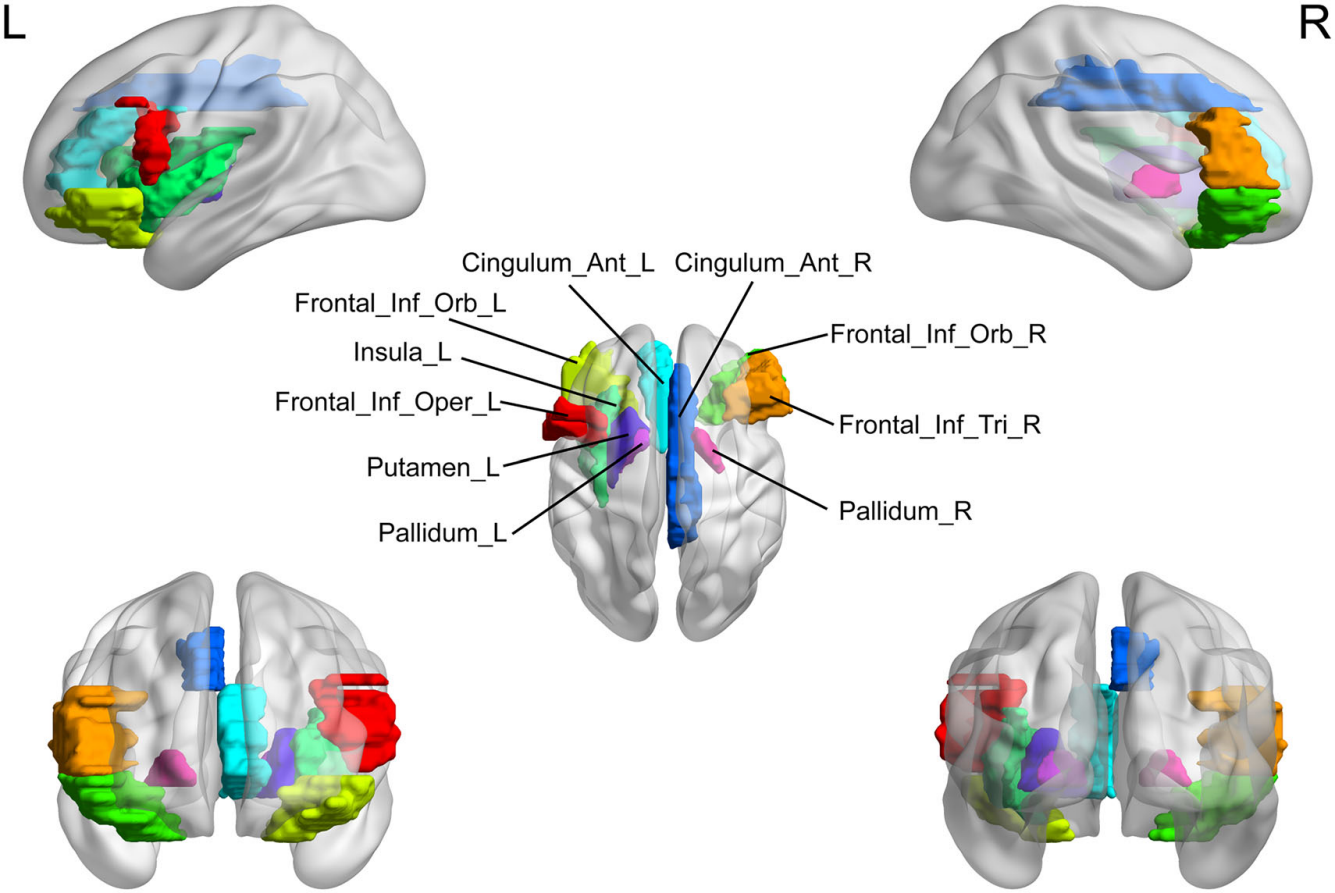
Brain regions with the significant differences in the nodal local efficiency (NLE) between the major depressive disorder (MDD) group and healthy controls (HC). HC, healthy controls; L, left; MDD, major depressive disorder; R, right.

**FIGURE 10:**
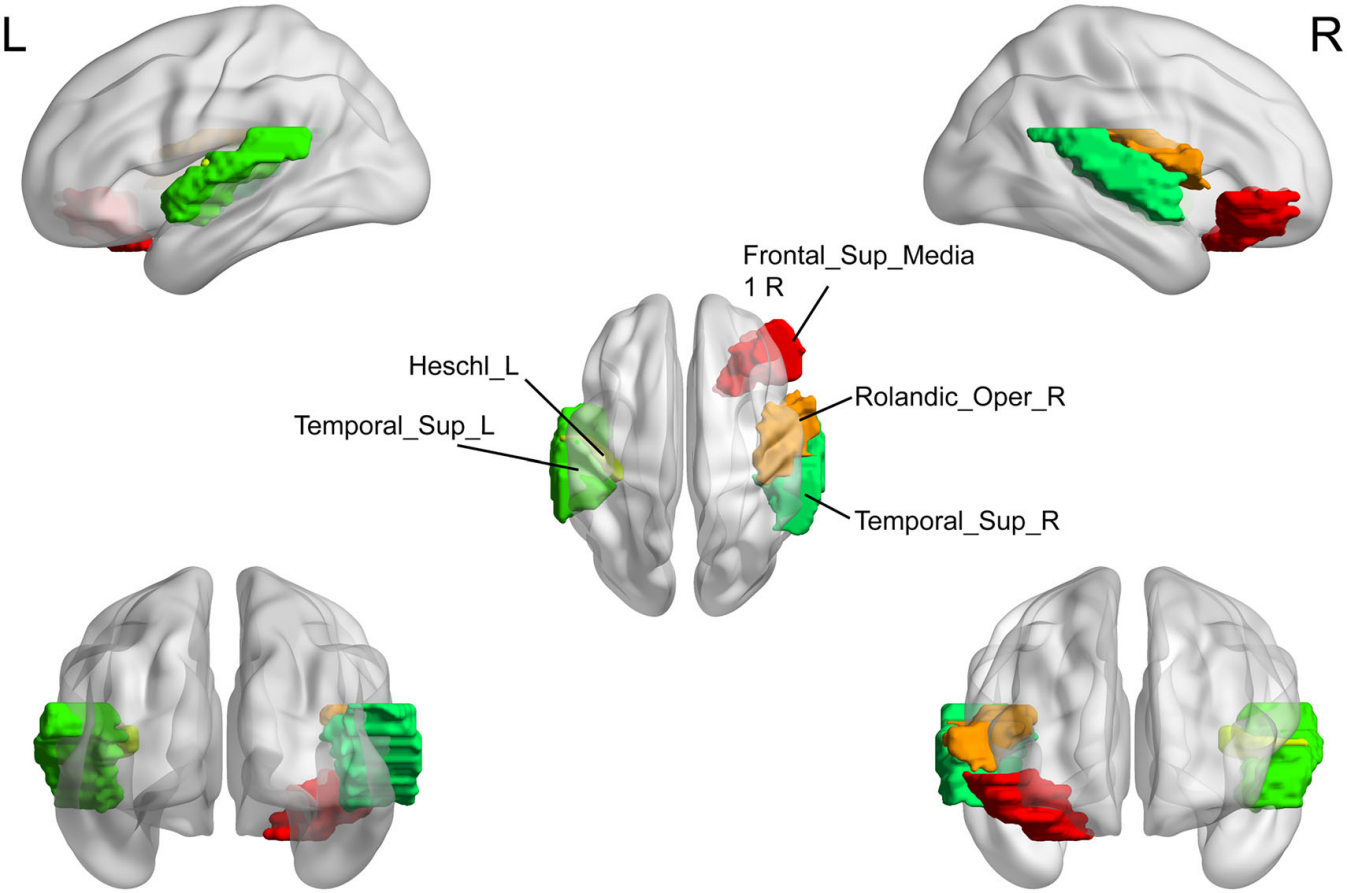
Brain regions with the significant differences in the nodal shortest path length (NLP) between the major depressive disorder (MDD) group and healthy controls (HC). HC, healthy controls; L, left; MDD, major depressive disorder; R, right.

Brain regions with significant differences in node topological properties between the MDD group and HC group were mainly located in the DMN, salience network (SAN), and cortical‐basal ganglia‐thalamo‐cortical circuit. Among them, the elevated nodal properties in adolescents with MDD compared to the HC group were primarily in the ACG.L (where ACG is anterior cingulate and paracingulate gyri), SFGmed.L (where SFGmed is medial superior frontal gyrus), SFGmed.R, ORBsupmed.L (where ORBsupmed is medial orbital superior frontal gyrus), ORBinf.R (where ORBinf is inferior frontal gyrus, orbital part), ROL.R (where ROL is rolandic operculum), HES.L (where HES is heschl gyrus), STG.L (where STG is superior temporal gyrus), and STG.R (*p *< 0.05) (Table 3; Figure 5, 6, 7, 8, 9, 10). In contrast, the brain regions with reduced nodal attributes were located in the HIP.R, HES.L, IFGoperc.L (where IFGoperc is opercular part of inferior frontal gyrus), INS.L (where INS is insula), DCG.R (where DCG is median cingulate and paracingulate gyri), PUT.L (where PUT is putamen lenticular nucleus), PUT.R, PAL.L (where PAL is pallidum lenticular nucleus), PAL.R, ORBinf.R, ROL.R, SMG.R, HES.L, STG.L, STG.R, IFGtriang.R, ORBinf.L, and ORBinf.R (*p* < 0.05) (Table 3; Figures 5, 6, 7, 8, 9, 10).

**TABLE 3:** Comparison of node attribute *p* values between adolescents with major depressive disorder (MDD) and healthy controls.

Brain region	BC	NCp	DC	NLE	NE	NLp
Frontal_Inf_Oper_L	—	0.04b	—	0.037b	—	—
Frontal_Inf_Tri_R	—	—	—	0.041b	—	—
Frontal_Inf_Orb_L	—	—	—	0.039b	—	—
Frontal_Inf_Orb_R	—	—	0.043b	0.037b	—	0.04a
Rolandic_Oper_R	—	—	0.019b	—	0.027b	0.025a
Frontal_Sup_Medial_L	—	—	0.036a	—	0.026a	—
Frontal_Sup_Medial_R	—	—	0.012a	—	0.011a	—
Frontal_Mid_Orb_L	—	—	—	—	0.049a	—
Insula_L	—	0.013b	—	0.035b	—	—
Cingulum_Ant_L	—	0.029a	—	0.029a	—	—
Cingulum_Mid_R	—	0.029b	—	0.023b	—	—
Hippocampus_R	0.042b	—	—	—	—	—
SupraMarginal_R	—	—	0.032b	—	—	—
Putamen_L	—	0.021b	—	0.032b	—	—
Putamen_R	—	0.017b	—	—	—	—
Pallidum_L	—	0.007b	—	0.002b	—	—
Pallidum_R	—	0.012b	—	0.006b	—	—
Heschl_L	0.045b	—	0.042b	—	0.046b	0.024a
Temporal_Sup_L	—	—	0.034b	—	0.044b	0.039a
Temporal_Sup_R	—	—	0.019b	—	0.033b	0.041a

*Note*: Statistical analysis was performed using a two independent samples *t*‐test, with *p *< 0.05. Group a: MDD > HC; Group b: MDD < HC; the difference between the two groups (“—”) was not statistically significant.

Abbreviation: BC, betweenness centrality; DC, node centrality; NCp, node clustering coefficient; NE, node efficiency; NLE, node local efficiency; NLp, node shortest path length.

### 3.4 Correlations Between the Aberrant Nodal Network Topology and Disease Severity

**FIGURE 11:**
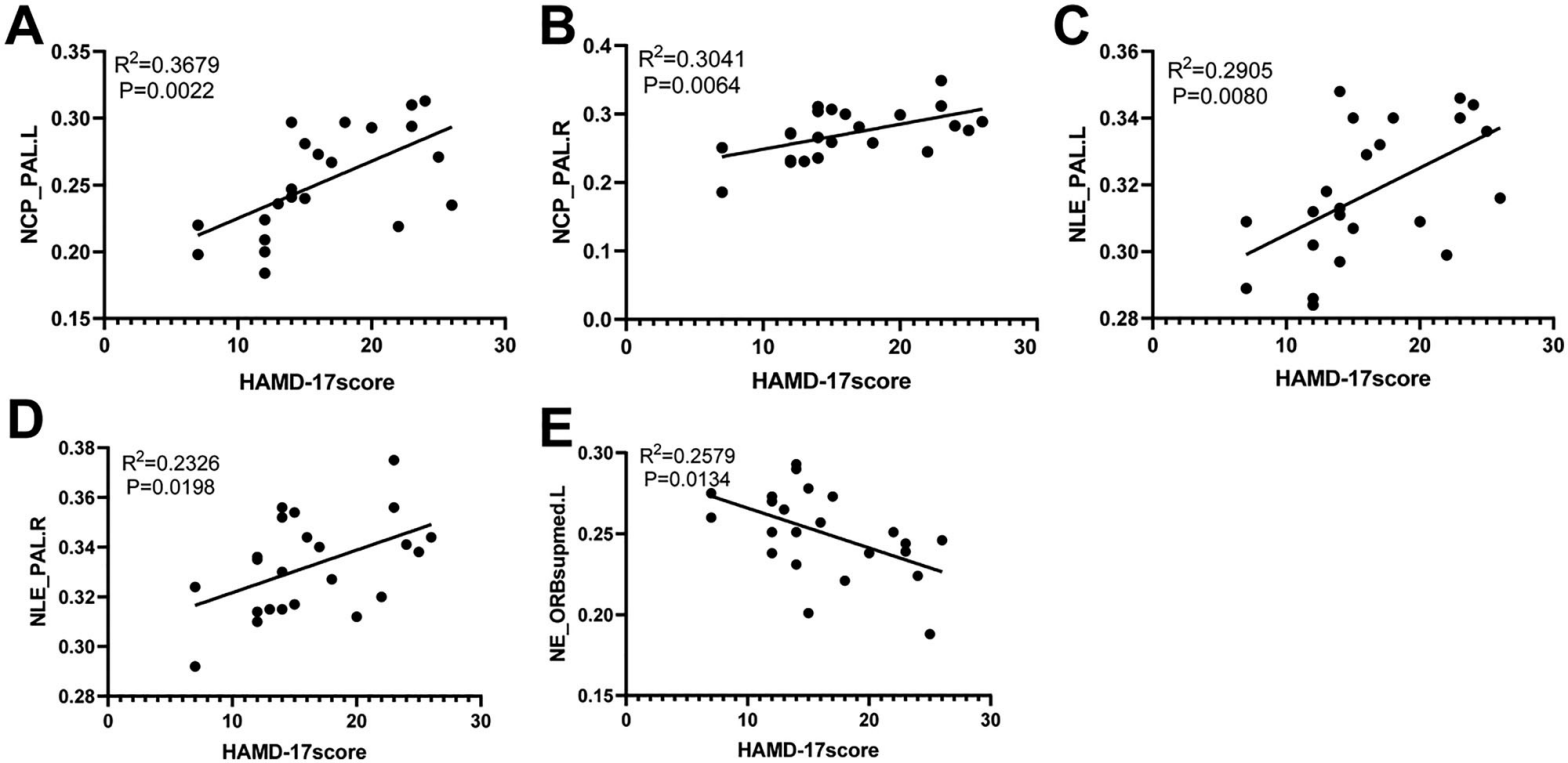
Correlation analysis of node attributes and clinical variables for abnormalities in the major depressive disorder (MDD) group. (A) Nodal clustering efficiency (NCp) in the left pallidum (PAL.L), (B) NCp in the right pallidum (PAL.R), (C) node local efficiency (NLE) in the left pallidum (PLA.L), and (D) NLE in the right pallidum (PAL.R) were positively associated with disease course severity. (E) Node efficiency (NE) in the superior frontal gyrus, medial orbital (ORBsupmed.L) was negatively associated with disease course severity (Pearson correlation).

We next performed correlation analysis between the node attributes of adolescents with MDD and HAMD‐17 scores. Interestingly, in the MDD group, the nodal Cp (NCp) of the PAL.L (*r* ^2^ = 0.37, *p *< 0.01) and PAL.R (*r* ^2^ = 0.31, *p *< 0.01) had significant positive correlations with the course of the disease. The nodal local efficiency (NLE) of (*r* ^2^ = 0.29, *p *< 0.01) and PAL.R (*r* ^2^ = 0.23, *p *= 0.02) was also positively correlated with the depression severity in the patients. In contrast, the NE of ORBsupmed.L was inversely associated with HAMD‐17 scores (*r* ^2^ = 0.26, *p* = 0.01) (Figure 11).

### 3.5 The Differentiation of Adolescents With MDD From HCs Using the Nodal Network Topological Properties

**FIGURE 12:**
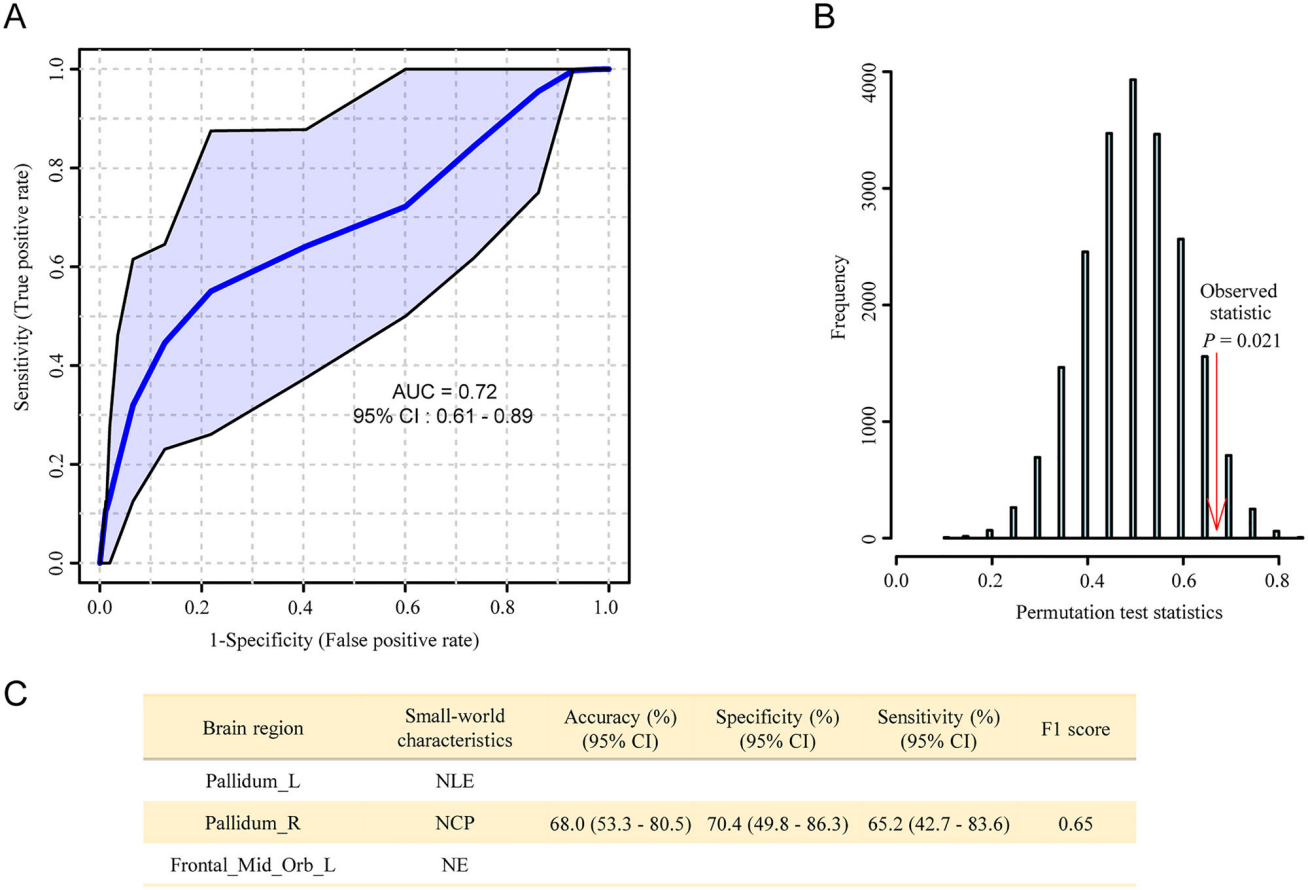
Receiver operating characteristic (ROC) analysis showed the differentiation of adolescents with major depressive disorder (MDD) from controls. (A) ROC curve analysis and area under the ROC (AUC). The nodal clustering efficiency (NCp) in the PAL.R, the node local efficiency (NLE) in the PAL.L, and the node efficiency (NE) in the ORBsupmed.L were used to build a classification model with the random forest algorithm. (B) The validation of the developed model by the permutation test (*n* = 1000). The *y*‐axis in this histogram does not represent the direct raw frequency count of the test statistics. Due to the large bin width used in the histogram, multiple distinct test statistic values from different permutations are grouped into the same bin. (C) The accuracy, specificity, and sensitivity of the developed model revealed by a 2 × 2 contingency analysis. NCp, nodal clustering efficiency; NE, node efficiency; NLE, node local efficiency; ORBsupmed, medial orbital of superior frontal gyrus; PAL, pallidum lenticular nucleus. *N* = 27 for healthy controls and 23 for adolescents with MDD.

The nodal network topological properties were ranked based on their mean decrease accuracy values in the RF algorithm (*n* = 1000 trees). A multivariate model with the NCp in the PAL.R, the NLE in the PAL.L and the NE in the ORBsupmed.L had the optimal performance for the differentiation of MDD patients from HCs with the area under the ROC curve (AUROC) of 0.71 (95% CI: 0.54–0.92). The model was further validated by the permutation test (*n* = 1000, *p* = 0.021). The accuracy, specificity, and sensitivity were 68%, 70.4%, and 65.2%, respectively. The F1 score was 0.65, which indicated a good classification model (Figure 12).

## 4 Discussion

Adolescent depression involves complex etiologies and mechanisms, often manifesting with subtle symptoms. This study identified abnormal small‐world properties in the brain functional networks of medication‐naïve adolescents experiencing their first episode of MDD. Our findings reveal that abnormal brain network topology in adolescent MDD mainly reflects reduced local information processing and diminished connectivity in key brain regions. These unique network characteristics provide crucial insights into the disorder's etiology. By integrating machine learning with knowledge‐based approaches, we developed a diagnostic model that differentiates MDD from healthy adolescents, achieving an AUROC exceeding 0.7 using distinct small‐world network features. If replicated, these findings could offer crucial clinical implications, providing objective imaging biomarkers to stratify adolescents and potentially prevent harm from delayed treatment.

Our research revealed that adolescents with MDD displayed a global brain functional network characterized by small‐world topology. This finding aligns with previous studies on adult MDD (Zhang et al. 2011; Leistedt et al. 2009), which suggests that the human brain functions as a small‐world network with high clustering coefficients and short path lengths (Lin et al. 2018). This network structure balances functional segregation and integration, where segregation refers to distinct brain regions handling specific tasks, and integration involves coordination and feedback between these regions during tasks (Huang et al. 2019).

We observed that reduced global Cp in adolescents with MDD is strongly associated with deficits in emotion regulation and executive function. A reduction in Cp reflects diminished local information processing, particularly in regions like the anterior cingulate cortex (ACC) and medial prefrontal cortex (MPFC), both linked to emotion regulation. The ACC is critical for resolving emotional conflicts and controlling responses, while the MPFC is closely linked to social cognition and self‐regulation of emotions (Etkin Egner, and Kalisch 2011; Ochsner and Gross 2005). Reduced Cp may cause functional abnormalities in these regions, impairing emotion regulation. In addition, executive function deficits are associated with structural changes in the PFC, affecting working memory, cognitive flexibility, and behavioral control (Levin et al. 2007). Therefore, these functional impairments in MDD patients may result from reduced efficiency in brain network information transmission.

Gender differences significantly influence brain network organization. Our study revealed that male adolescents with MDD exhibited significantly lower Cp compared to females, suggesting that males may be more vulnerable to information processing and emotional regulation. Males and females engage distinct brain regions for emotion processing, with females relying more on the limbic system and males on the PFC (Stevens and Hamann 2012). This divergence may explain why males with lower global Cp exhibit more severe cognitive and emotional symptoms. In addition, male patient's brain functional networks are more susceptible to random disruptions, indicating lower structural robustness and adaptability, potentially making them more vulnerable to external stressors and worsening clinical symptoms (Lai and Pasternak 2015). In summary, gender differences in adolescent MDD brain network alterations suggest that reduced Cp is closely linked to deficits in emotion regulation and executive function, providing new insights into the clinical manifestations of adolescent depression.

Adolescence is a pivotal period marked by significant neurodevelopmental changes, especially in networks tied to emotion regulation and cognitive function. This maturation stage involves gray matter reduction, white matter increases, and dynamic adjustments in functional integration and segregation within neural networks (Somerville and Casey 2010). These changes increase adolescent's susceptibility to external stressors, leading to vulnerabilities in emotional regulation and exacerbating the pathophysiological processes of depression (Blakemore 2012). In adolescents, brain regions involved in emotion regulation, including the PFC and limbic system (amygdala, hippocampus), are not yet fully matured, which may hinder their ability to cope with emotions and stress (Casey, Jones, and Hare 2008). Studies show that adolescents with depression exhibit weaker effective connectivity between the DMN and SAN, possibly reflecting abnormalities in emotion regulation and self‐awareness (Willinger et al. 2024). The ongoing maturation of the adolescent brain may alter the topology of functional networks, leading to different patterns compared to adult MDD.As the brain gradually develops more efficient connectivity, adolescents may become more susceptible to external factors like social pressure, potentially triggering MDD (Davey, Yücel, and Allen 2008). During this period, accelerated synaptic pruning and white matter myelination affect the strength and efficiency of connections between brain regions (Luna and Sweeney 2004). These developmental processes shape the organization and interaction of brain networks like the DMN, SN, and executive control network, leading to differences in the presentation of adolescent MDD compared to adult MDD (Pfeifer and Allen 2012). In addition, immature emotional regulation and cognitive control systems in the adolescent brain may exacerbate MDD symptoms, resulting in more pronounced emotional fluctuations and cognitive deficits (Cullen et al. 2014). Although many brain network changes identified in this study align with adult MDD research, the adolescent brain remains in a developmental phase, and its functional integration and segregation may not be fully matured, leading to differences in network changes compared to adult MDD.

In this study, we report for the first time a reduction in NCp and NLE in the globus pallidus, both of which are significantly correlated with disease severity. These findings suggest reduced local information processing capabilities and connectivity with adjacent nodes in the globus pallidus in depressed children, potentially contributing to observed abnormalities in somatic and sensorimotor function. In addition, reduced nodal characteristics in the nucleus accumbens and globus pallidus further suggest potential basal ganglia dysfunction in adolescent MDD patients.

Despite identifying these significant brain network abnormalities, this study has several limitations. First, the small sample size may limit the statistical power of our findings, especially in the gender subgroup analyses. We conducted a power analysis and determined that 30 participants per group would provide a statistical power of 0.8 (*β* = 0.2) to detect effects at a Type I error rate of 0.05 (*α* = 0.05) (Button et al. 2013). Second, although the machine learning classifier achieved an AUC of over 0.7, the small sample size may heighten the risk of overfitting. Thus, future research needs to validate these classification results in larger independent samples to ensure their clinical applicability (Varoquaux 2018; Pereira, Mitchell, and Botvinick 2009). Finally, longitudinal studies are needed to examine dynamic changes in brain networks in adolescents with depression, which will help clarify the role of these changes in disease progression.

## 5 Conclusion

In this study, we utilized a graph theory approach to analyze the functional brain networks of adolescents with first episode, untreated MDD. Our findings demonstrated that both MDD patients and control groups exhibited small‐world network properties, indicating an overall balance between local and global information processing. However, MDD patients showed impairments in local information processing, particularly in the DMN, SAN, and basal ganglia regions. These areas displayed significant abnormalities, suggesting widespread dysfunction across multiple brain networks in adolescents with MDD. Specifically, the bilateral globus pallidus showed a positive correlation with the severity of depressive symptoms, while the left intraorbital superior frontal gyrus was negatively correlated with disease duration. Importantly, we developed a new model using the NCp value of the right pallidum, which demonstrated good accuracy in distinguishing adolescent MDD patients from HCs. This study provides novel imaging evidence that can aid in the objective diagnosis of adolescent MDD and offers insights into the underlying neural mechanisms contributing to the disorder's pathophysiology.

## Author Contributions

**Song Cheng**: writing–original draft, methodology, software. **Henry H. Y. Tong**: writing–original draft, software, data curation. **Chaoqing Zhang**: investigation. **Lingyu Jian**: investigation. **Junjun Ji**: investigation. **Ting Li**: investigation. **Yu Wang**: investigation. **Junfeng Li**: funding acquisition, conceptualization, methodology, writing–review and editing. **Kefeng Li**: conceptualization, methodology, writing–original draft, writing–review and editing.

## Conflicts of Interest

The authors declare no conflicts of interest.

### Peer Review

The peer review history for this article is available at https://publons.com/publon/10.1002/brb3.70323

## Data Availability

Our data is still under further study, and we will make the data publicly available when the full study is completed.

## References

[brb370323-bib-0001] Baller, E. B. , A. N. Kaczkurkin , A. Sotiras , et al. 2021. “Neurocognitive and Functional Heterogeneity in Depressed Youth.” Neuropsychopharmacology 46, no. 4: 783–790. 10.1038/s41386-020-00871-w. 33007777 PMC8027806

[brb370323-bib-0002] Bella, M. E. , R. A. Fernández , and J. M. Willington . 2010. “Depression and Conduct Disorder Are the Most Frequent Pathologies in Child and Adolescent Suicide Attempt.” Archivos Argentinos de Pediatría 108, no. 2: 124–129. 10.1590/s0325-00752010000200006. 20467707

[brb370323-bib-0003] Blakemore, S. J. 2012. “Imaging Brain Development: The Adolescent Brain.” NeuroImage 61, no. 2: 397–406. 10.1016/j.neuroimage.2011.11.080. 22178817

[brb370323-bib-0004] Bullmore, E. , and O. Sporns . 2009. “Complex Brain Networks: Graph Theoretical Analysis of Structural and Functional Systems.” Nature Reviews Neuroscience 10, no. 3: 186–198. 10.1038/nrn2575. 19190637

[brb370323-bib-0005] Button, K. S. , J. P. A. Ioannidis , C. Mokrysz , et al. 2013. “Power Failure: Why Small Sample Size Undermines the Reliability of Neuroscience.” Nature Reviews Neuroscience 14, no. 5: 365–376. 10.1038/nrn3475. 23571845

[brb370323-bib-0006] Casey, B. J. , R. M. Jones , and T. A. Hare . 2008. “The Adolescent Brain.” Annals of the New York Academy of Sciences 1124, no. 1: 111–126. 10.1196/annals.1440.010. 18400927 PMC2475802

[brb370323-bib-0007] Chen, M.‐H. , W.‐C. Chang , W.‐C. Lin , et al. 2020. “Functional Dysconnectivity of Frontal Cortex to Striatum Predicts Ketamine Infusion Response in Treatment‐Resistant Depression.” International Journal of Neuropsychopharmacology 23, no. 10: 791–798. 10.1093/ijnp/pyaa056. 32726408 PMC7770518

[brb370323-bib-0008] Cullen, K. R. , M. K. Westlund , B. Klimes‐Dougan , et al. 2014. “Abnormal Amygdala Resting‐State Functional Connectivity in Adolescent Depression.” JAMA Psychiatry 71, no. 10: 1138–1147. 10.1001/jamapsychiatry.2014.1087. 25133665 PMC4378862

[brb370323-bib-0009] Davey, C. G. , M. Yücel , and N. B. Allen . 2008. “The Emergence of Depression in Adolescence: Development of the Prefrontal Cortex and the Representation of Reward.” Neuroscience & Biobehavioral Reviews 32, no. 1: 1–19. 10.1016/j.neubiorev.2007.04.016. 17570526

[brb370323-bib-0010] Dias‐Ferreira, E. , J. C. Sousa , I. Melo , et al. 2009. “Chronic Stress Causes Frontostriatal Reorganization and Affects Decision‐Making.” Science 325, no. 5940: 621–625. 10.1126/science.1171203. 19644122

[brb370323-bib-0011] Etkin, A. , T. Egner , and R. Kalisch . 2011. “Emotional Regulation in Anxiety and Depression: Neural Mechanisms.” Nature Reviews Neuroscience 12, no. 12: 846–857. 10.1038/nrn3143.

[brb370323-bib-0012] Fateh, A. A. , Z. Long , X. Duan , et al. 2019. “Hippocampal Functional Connectivity‐Based Discrimination Between Bipolar and Major Depressive Disorders.” Psychiatry Research: Neuroimaging 284: 53–60. 10.1016/j.pscychresns.2019.01.004. 30684896

[brb370323-bib-0013] Gourley, S. L. , A. M. Swanson , and A. J. Koleske . 2013. “Corticosteroid‐Induced Neural Remodeling Predicts Behavioral Vulnerability and Resilience.” Journal of Neuroscience 33, no. 7: 3107–3112. 10.1523/jneurosci.2138-12.2013. 23407965 PMC3711631

[brb370323-bib-0014] Gunnell, D. , J. Kidger , and H. Elvidge . 2018. “Adolescent Mental Health in Crisis.” BMJ 361: k2608. 10.1136/bmj.k2608. 29921659

[brb370323-bib-0015] He, Y. , J. Wang , L. Wang , et al. 2009. “Uncovering Intrinsic Modular Organization of Spontaneous Brain Activity in Humans.” PLoS One 4, no. 4: e5226. 10.1371/journal.pone.0005226. 19381298 PMC2668183

[brb370323-bib-0016] Holt, R. J. , J. M. Graham , K. J. Whitaker , et al. 2016. “Functional MRI of Emotional Memory in Adolescent Depression.” Developmental Cognitive Neuroscience 19: 31–41. 10.1016/j.dcn.2015.12.013. 26802367 PMC4913558

[brb370323-bib-0017] Huang, Y. , Y. Liu , D. Zhao , et al. 2019. “Small‐World Properties of the Whole‐Brain Functional Networks in Patients With Obstructive Sleep Apnea‐Hypopnea Syndrome.” Sleep Medicine 62: 53–58. 10.1016/j.sleep.2018.08.037. 31557687

[brb370323-bib-0018] Jamieson, A. J. , B. J. Harrison , A. Razi , and C. G. Davey . 2022. “Rostral Anterior Cingulate Network Effective Connectivity in Depressed Adolescents and Associations With Treatment Response in a Randomized Controlled Trial.” Neuropsychopharmacology 47, no. 6: 1240–1248. 34782701 10.1038/s41386-021-01214-z PMC9018815

[brb370323-bib-0019] Kaiser, R. H. , J. R. Andrews‐Hanna , T. D. Wager , and D. A. Pizzagalli . 2015. “Large‐Scale Network Dysfunction in Major Depressive Disorder: A Meta‐Analysis of Resting‐State Functional Connectivity.” JAMA Psychiatry 72, no. 6: 603–611. 10.1001/jamapsychiatry.2015.0071. 25785575 PMC4456260

[brb370323-bib-0020] Kraus, C. , A. Mkrtchian , B. Kadriu , A. C. Nugent , C. A. Zarate , and J. W. Evans . 2020. “Evaluating Global Brain Connectivity as an Imaging Marker for Depression: Influence of Preprocessing Strategies and Placebo‐Controlled Ketamine Treatment.” Neuropsychopharmacology 45, no. 6: 982–989. 10.1038/s41386-020-0624-0. 31995812 PMC7162890

[brb370323-bib-0021] Lai, M. C. , and O. Pasternak . 2015. “Sex Differences in the Structural Connectome of the Human Brain.” Proceedings of the National Academy of Sciences of the United States of America 112, no. 20: 6495–6500. 10.1073/pnas.1505652112. PMC3896179 24297904

[brb370323-bib-0022] Leistedt, S. J. J. , N. Coumans , M. Dumont , J.‐P. Lanquart , C. J. Stam , and P. Linkowski . 2009. “Altered Sleep Brain Functional Connectivity in Acutely Depressed Patients.” Human Brain Mapping 30, no. 7: 2207–2219. 10.1002/hbm.20662. 18937282 PMC6870637

[brb370323-bib-0023] Levin, R. L. , W. Heller , A. Mohanty , J. D. Herrington , and G. A. Miller . 2007. “Cognitive Deficits in Depression and Functional Specificity of Regional Brain Activity.” Cognitive Therapy and Research 31, no. 2: 211–233. 10.1007/s10608-006-9128-z.

[brb370323-bib-0024] Li, J. , Z. Yang , H. Qiu , et al. 2020. “Anxiety and Depression Among General Population in China at the Peak of the COVID‐19 Epidemic.” World Psychiatry 19, no. 2: 249–250. 10.1002/wps.20758. 32394560 PMC7214959

[brb370323-bib-0025] Lin, L. , Z. Fu , C. Jin , M. Tian , and S. Wu . 2018. “Small‐World Indices via Network Efficiency for Brain Networks From Diffusion MRI.” Experimental Brain Research 236, no. 10: 2677–2689. 10.1007/s00221-018-5326-z. 29980823

[brb370323-bib-0026] Liston, C. , M. M. Miller , D. S. Goldwater , et al. 2006. “Stress‐Induced Alterations in Prefrontal Cortical Dendritic Morphology Predict Selective Impairments in Perceptual Attentional Set‐Shifting.” Journal of Neuroscience 26, no. 30: 7870–7874. 10.1523/jneurosci.1184-06.2006. 16870732 PMC6674229

[brb370323-bib-0027] Luczynski, P. , L. Moquin , and A. Gratton . 2015. “Chronic Stress Alters the Dendritic Morphology of Callosal Neurons and the Acute Glutamate Stress Response in the Rat Medial Prefrontal Cortex.” Stress 18, no. 6: 654–667. 10.3109/10253890.2015.1073256. 26364921

[brb370323-bib-0028] Luna, B. , and J. A. Sweeney . 2004. “The Emergence of Collaborative Brain Function: fMRI Studies of the Development of Response Inhibition.” Annals of the New York Academy of Sciences 1021, no. 1: 296–309. 10.1196/annals.1308.035. 15251900

[brb370323-bib-0029] Mulders, P. C. , P. F. van Eijndhoven , A. H. Schene , C. F. Beckmann , and I. Tendolkar . 2015. “Resting‐State Functional Connectivity in Major Depressive Disorder: A Review.” Neuroscience and Biobehavioral Reviews 56: 330–344. 10.1016/j.neubiorev.2015.07.014. 26234819

[brb370323-bib-0030] Nelson, C. J. , and S. Bonner . 2021. “Neuronal Graphs: A Graph Theory Primer for Microscopic, Functional Networks of Neurons Recorded by Calcium Imaging.” Frontiers in Neural Circuits 15: 662882. 10.3389/fncir.2021.662882. 34177469 PMC8222695

[brb370323-bib-0031] Ochsner, K. N. , and J. J. Gross . 2005. “The Cognitive Control of Emotion.” Trends in Cognitive Sciences 9, no. 5: 242–249. 10.1016/j.tics.2005.03.010. 15866151

[brb370323-bib-0032] Pereira, F. , T. Mitchell , and M. Botvinick . 2009. “Machine Learning Classifiers and fMRI: A Tutorial Overview.” NeuroImage 45, no. 1: S199–S209. 10.1016/j.neuroimage.2008.11.007. 19070668 PMC2892746

[brb370323-bib-0033] Pfeifer, J. H. , and N. B. Allen . 2012. “Arrested Development? Reconsidering Dual‐Systems Models of Brain Function in Adolescence and Disorders.” Trends in Cognitive Sciences 16, no. 6: 322–329. 10.1016/j.tics.2012.04.011. 22613872 PMC3711850

[brb370323-bib-0034] Qiu, H. , and J. Li . 2018. “Major Depressive Disorder and Magnetic Resonance Imaging: A Mini‐Review of Recent Progress.” Current Pharmaceutical Design 24, no. 24: 2524–2529. 10.2174/1381612824666180727111651. 30051779

[brb370323-bib-0035] Smith, K. 2014. “Mental Health: A World of Depression.” Nature 515, no. 7526: 181. 10.1038/515180a. 25391942

[brb370323-bib-0036] Somerville, L. H. , and B. J. Casey . 2010. “Developmental Neurobiology of Cognitive Control and Motivational Systems.” Current Opinion in Neurobiology 20, no. 2: 236–241. 10.1016/j.conb.2010.01.006. 20167473 PMC3014528

[brb370323-bib-0037] Stevens, J. S. , and S. Hamann . 2012. “Sex Differences in Brain Activation to Emotional Stimuli: A Meta‐Analysis of Neuroimaging Studies.” Neuropsychologia 50, no. 7: 1578–1593. 10.1016/j.neuropsychologia.2012.03.011. 22450197

[brb370323-bib-0038] Uher, R. , J. L. Payne , B. Pavlova , and R. H. Perlis . 2014. “Major Depressive Disorder in DSM‐5: Implications for Clinical Practice and Research of Changes From DSM‐IV.” Depression and Anxiety 31, no. 6: 459–471. 10.1002/da.22217. 24272961

[brb370323-bib-0039] Varoquaux, G. 2018. “Cross‐Validation Failure: Small Sample Sizes Lead to Large Error Bars.” Neuroimage 180: 68–77. 10.1016/j.neuroimage.2017.06.061. 28655633

[brb370323-bib-0040] Willinger, D. , I. Häberling , I. Ilioska , G. Berger , S. Walitza , and S. Brem . 2024. “Weakened Effective Connectivity Between Salience Network and Default Mode Network During Resting State in Adolescent Depression.” Frontiers in Psychiatry 15: 1386984. 38638415 10.3389/fpsyt.2024.1386984 PMC11024787

[brb370323-bib-0041] Yang, Z. , L. Jian , H. Qiu , et al. 2021. “Understanding Complex Functional Wiring Patterns in Major Depressive Disorder Through Brain Functional Connectome.” Translational Psychiatry 11, no. 1: 526. 10.1038/s41398-021-01646-7. 34645783 PMC8513388

[brb370323-bib-0042] Yu, H. , F. Li , T. Wu , et al. 2018. “Functional Brain Abnormalities in Major Depressive Disorder Using the Hilbert‐Huang Transform.” Brain Imaging and Behavior 12: 1556–1568. 10.1007/s11682-017-9816-6. 29427063

[brb370323-bib-0043] Yu, M. , K. A. Linn , R. T. Shinohara , et al. 2019. “Childhood Trauma History Is Linked to Abnormal Brain Connectivity in Major Depression.” Proceedings of the National Academy of Sciences of the United States of America 116, no. 17: 8582–8590. 10.1073/pnas.1900801116. 30962366 PMC6486762

[brb370323-bib-0044] Zhang, J. , J. Wang , Q. Wu , et al. 2011. “Disrupted Brain Connectivity Networks in Drug‐Naive, First‐Episode Major Depressive Disorder.” Biological Psychiatry 70, no. 4: 334–342. 10.1016/j.biopsych.2011.05.018. 21791259

[brb370323-bib-0045] Zhao, Y.‐J. , M.‐Y. Du , X.‐Q. Huang , et al. 2014. “Brain Grey Matter Abnormalities in Medication‐Free Patients With Major Depressive Disorder: A Meta‐Analysis.” Psychological Medicine 44, no. 14: 2927–2937. 10.1017/s0033291714000518. 25065859

[brb370323-bib-0046] Zheng, R. , Y. Chen , Y.u Jiang , et al. 2022. “Abnormal Dynamic Functional Connectivity in First‐Episode, Drug‐Naïve Adolescents With Major Depressive Disorder.” Journal of Neuroscience Research 100, no. 7: 1463–1475. 10.1002/jnr.25047. 35393711

